# Synovial sarcoma of the maxilla: A challenging diagnostic case report and literature review

**DOI:** 10.1002/ccr3.8254

**Published:** 2023-11-25

**Authors:** Nasrollah Saghravanian, Amin Rahpeyma, Mahsa Ghorbani, Pooya Saeedi

**Affiliations:** ^1^ Oral and Maxillofacial Disease Research Center, School of Dentistry Mashhad University of Medical Sciences Mashhad Iran; ^2^ School of Dentistry Mashhad University of Medical Sciences Mashhad Iran

**Keywords:** case report, head and neck, maxilla, synovial sarcoma

## Abstract

Synovial sarcoma, a malignant mesenchymal tumor, is primarily associated with the extremities. Nevertheless, its appearance within the head and neck region, particularly in the maxillary area, is remarkably rare. This rarity underscores the significance of each case in unraveling the complexities of its behavior and management strategies. The core focus of this research is a detailed case report involving a 6‐year‐old female patient who presented with a conspicuous swelling in the left posterior maxilla. Subsequent incisional biopsy led to microscopic identification of malignant spindle cell proliferation, marked by dysplastic changes, and abundant mitoses. Immunohistochemical (IHC) analysis demonstrated negative reactivity for neural and muscular markers, while positive expression of Vimentin, Bcl‐2, and TLE1. These morphological and IHC findings coalesced to definitively diagnose synovial sarcoma, substantiated by a notable 40% Ki67 proliferative index. The chosen treatment strategy encompassed surgery and radiotherapy, which yielded successful outcomes, with no recurrence observed during the one‐year follow‐up period. Beyond the specific case, this article undertakes a review of existing literature, meticulously analyzing nine similar cases reported in scholarly sources.

## Graphical abstract

Microscopic examination with hematoxylin and eosin staining.

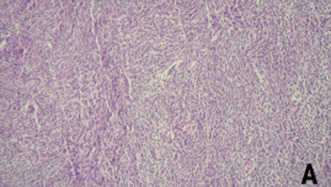

## 1 INTRODUCTION

Synovial sarcoma is a rare type of spindle cell tumor that constitutes approximately 10% of all soft tissue sarcomas.^1^ Despite its name, which originates from the microscopic resemblance to developing synovium, the exact origin of this tumor remains unknown. Sarcomas in the head and neck region are exceptionally uncommon, accounting for merely 1% of all primary tumors in this area and 4%–10% of sarcomas overall.^2^, ^3^ The majority of head and neck sarcomas (around 80%) arise from soft tissues, while only a minority (20%) originate from bone or cartilage.^2^, ^4^ These tumors originate from mesenchymal cells, encompassing a diverse group that can arise from various tissues such as bone, cartilage, muscle, fat, blood vessels, and nerves.^3^

Synovial sarcoma exhibits a higher prevalence in the third decade of life, primarily affecting teenagers and young adults between the ages of 15 and 40.^5^ The occurrence of synovial sarcoma in the maxillary region poses a distinct diagnostic challenge owing to its rare incidence. This challenge is particularly pronounced when the tumor is in its early stages, as it often exhibits slow growth and nonspecific symptoms, further complicating the diagnostic process.^6^ The complex anatomy of the head and neck region also poses limitations in achieving wide surgical margins, which contributes to increased local recurrence rates and poorer disease‐specific survival compared to sarcomas in other anatomical sites.^7^ Therefore, complete resection is considered the optimal treatment approach for synovial sarcoma.^8^

In this article, we present a challenging diagnostic case report of synovial sarcoma in the maxilla. We aim to shed light on the intricacies of diagnosing and managing this rare condition, emphasizing the importance of early detection and comprehensive surgical intervention for improved patient outcomes.

## 2 CASE PRESENTATION

In September 2021, a 6‐year‐old female patient presented to the Maxillofacial Surgery Department of Mashhad University of Medical Sciences with a complaint of swelling at the left posterior sites of the maxilla. The patient's medical history was unremarkable. Upon clinical examination, a soft, darker‐colored lesion was observed in the buccal and palatal regions, extending from tooth E to approximately the midline. The macroscopic examination revealed a large piece of soft, tumoral tissue along with multiple shapeless soft, and jelly‐like pieces. The largest piece measured 30 × 22 × 8 mm with an irregular surface, while the smaller pieces collectively measured 7 × 22 × 32 mm. An incisional biopsy was performed, and a soft tissue specimen was sent to the pathologist for further evaluation. Microscopic examination of serial sections from the submitted sample revealed a malignant proliferation of dysplastic changes and multiple mitoses of spindle cells. Immunohistochemical (IHC) analysis showed negativity for neural, muscular, plasma cell, and epithelial markers, including Myogenin, SMA, SOX10, CD34, and EMA. However, the tumor exhibited positive expression of Vimentin, Bcl‐2, and particularly TLE1 markers.

**FIGURE 1:**
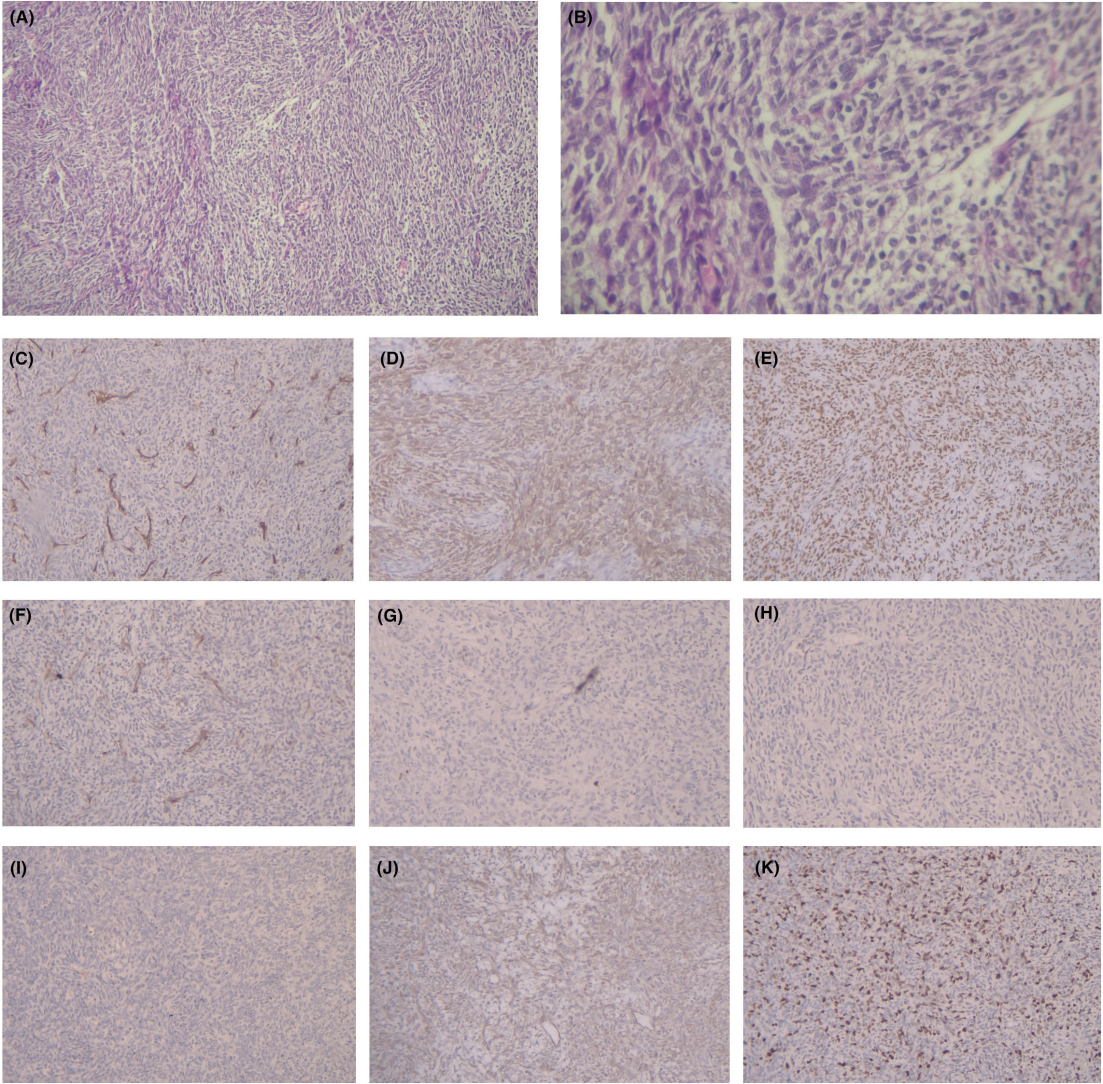
Microscopic examination and immunohistochemical analysis. (A, B) Hematoxylin and eosin staining. (C) Negative CD34 staining. (D) Positive Bcl‐2 staining. (E) Positive TLE1 staining. (F) Negative SMA staining. (G) Nnegative myogenin staining. (H) Negative EMA staining. (I) Negative SOX10 staining. (J) Positive Vimentin staining. (K) Ki67 staining demonstrating a 40% proliferation index.

Based on the morphologic and IHC findings, a diagnosis of synovial sarcoma was established and the diagnosis was further supported by a 40% Ki67 proliferative index. Figure 1 illustrates the hematoxylin and eosin staining and IHC analysis of the synovial sarcoma sample. The patient was referred for further evaluation and treatment planning to determine the optimal management strategy for the synovial sarcoma.

Furthermore, the treatment strategy for synovial sarcoma was meticulously devised. Given the complexity of the tumor's location, a surgical approach was chosen as the primary intervention. The surgical procedure aimed at achieving complete resection with clear margins, thereby minimizing the risk of local recurrence. However, due to the challenging anatomical constraints of the head and neck region, obtaining wide surgical margins can be intricate. To enhance the treatment's effectiveness and address potential residual microscopic disease, a multimodal approach was adopted. This encompassed a combination of surgery and adjuvant radiotherapy. The integration of radiotherapy aimed to target any remaining cancer cells while also acting as a preventive measure against possible recurrence. This comprehensive treatment approach was tailored to not only address the immediate tumor but also to ensure long‐term disease control and optimal patient outcomes.

After the completion of treatment, meticulous follow‐up was conducted to monitor the patient's progress. The patient underwent regular clinical assessments and imaging studies. Encouragingly, at the one‐year follow‐up mark, no evidence of disease was observed.

## 3 DISCUSSION

Synovial sarcoma is a rare malignancy primarily affecting the extremities, with its occurrence in the maxillofacial region being exceptionally rare. The unique anatomical location of synovial sarcoma in the maxillofacial region poses significant diagnostic and therapeutic challenges.^9^ In our case, a 6‐year‐old female presented with a swelling in the left posterior sites of the maxilla. The histopathological examination revealed morphologic features consistent with synovial sarcoma. The confirmation of synovial sarcoma was further supported by IHC analysis, which showed positivity for vimentin, bcl2, and Tle1 markers. Despite ongoing debates regarding the specificity and diagnostic significance of the Tle1 staining test, a systematic review has validated its sensitivity and specificity as a marker for synovial sarcoma. Additionally, it is suggested that this marker might hold direct relevance to the disease's pathophysiology.^10^ The treatment approach involved surgery for complete resection followed by adjuvant radiotherapy to prevent recurrence. After treatment, regular follow‐up assessments and imaging were conducted and at the one‐year follow‐up, no evidence of disease was observed. Synovial sarcoma is characterized by its biphasic histology, comprising epithelial and spindle cell components. However, our case demonstrated a predominantly spindle cell pattern. This variant, known as monophasic synovial sarcoma, is less common but has been reported in the literature.^11^, ^12^ The diagnosis of synovial sarcoma in the maxillofacial region requires careful consideration of the histopathological features, along with the characteristic IHC profile. Similar cases of synovial sarcoma in the maxillary region have been reported in the literature.

We meticulously reviewed a collection of nine cases of synovial sarcoma in the maxillary region from the literature,^9^, ^13^, ^14^, ^15^, ^16^, ^17^, ^18^ all of which are concisely summarized in Table 1. The patient age range spanned from 26 to 79 years, with an average age of 46.5 years and a male‐to‐female ratio of 5:4. However, our case adds a distinctive facet as we detail an exceptionally rare occurrence in a 6‐year‐old girl. Among these nine cases, three manifested in the parotid gland,^13^ four in the maxillary and paranasal sinuses,^9^, ^14^, ^16^, ^17^ one in the nasal septum,^18^ and one in the maxilla^15^—resembling our presented case. Surgical intervention was universally applied, with six instances followed by adjuvant radiotherapy,^9^, ^13^, ^14^, ^16^, ^17^ one with post‐recurrence radiotherapy,^13^ and three receiving chemotherapy as supplementary treatment.^14^, ^15^, ^16^ Another single case was managed solely with surgery,^18^ while the remaining eight involved a combination of surgery, radiotherapy, and/or chemotherapy. Correspondingly, our case underwent surgery followed by adjuvant radiotherapy. Among the three cases that underwent chemotherapy, Maxymiw and Wood^15^ elucidated the regimen (three cycles of doxorubicin, cyclophosphamide, and vincristine), while Saito et al.^16^ adopted three courses of ifosfamide and pirarubicin; both regimens achieved tumor mass reduction. However, it's important to note that in the case presented by Lin et al.,^14^ there was a lack of specific details regarding the chemotherapy protocol. The average follow‐up span extended to 28 months, with two out of the nine cases dying within 8 and 96 months. Maxymiw and Wood's case resulted in death due to neuropathy and multiple system failure,^15^ while Kartha and Bumpous's case did not specify the cause of death.^13^ Additionally, three cases experienced recurrence within 1 month to 4 years,^13^, ^14^ though they remained alive. Conversely, other cases showed no evidence of disease during the follow‐up period.

**TABLE 1:** Summary of Synovial sarcoma cases in the maxillary region.

Author, Year	Age [years], Sex	Site	Size [mm]	Treatment	Outcome, Follow‐up period
Maxymiw, 1990	32, F	Maxilla	NR	Surgery, CTx	Died, 8 months (recurred in 3 months)
Kartha, 2002	26, M	Parotid gland	3 × 2 × 0.5	Surgery, RTx after recurrence	Died, 96 months (recurred in 12 months)
	32, M	Parotid gland	60 × 40 × 40	Surgery, RTx	AWED, 1 month
	38, F	Parotid gland	25 × 18 × 15 & 20 × 17 × 15	Surgery, RTx	AWED, 48 months
Sun, 2003	54, M	Maxillary, ethmoid, and frontal sinuses, nasal cavity	NR	Surgery, RTx	NED, 45 months
Yildirim, 2005	52, F	Nasal septum	15 × 15	Surgery	NED, 18 months
Saito, 2018	53, M	Maxillary, ethmoid, and frontal sinuses, pterygopalatine and infratemporal fossa	50 × 58	Surgery, RTx, CTx	NED, 12 months
Lin, 2021	79, F	Maxillary, ethmoid, and frontal sinuses, nasal cavity	NR	Surgery, RTx, CTx	AWED, 2 years
Hannoun, 2021	53, M	Maxillary sinus, nasal cavity, hard palate, eustachian tube, pterygopalatine fossa	38 × 28 × 42	Surgery, RTx	NED, 5 months
Present case	6, F	Maxilla	30 × 22 × 8	Surgery, RTx	NED, 1 year

Abbreviations: AWED, alive with evidence of disease; CTx, chemotherapy; F, female; M, male; NED, no evidence of disease; NR, not recorded; RTx, radiotherapy.

It is important to note that the case we present is a singular case report, and its follow‐up period remains relatively short. Analogous to synovial sarcomas affecting extremities, metastasis or local recurrence can manifest after a certain interval. Consequently, we are closely monitoring the progress of our current patient.

From the patient's perspective, undergoing treatment for synovial sarcoma in the maxillary region was a complex journey, particularly given the young age at diagnosis, which was emotionally challenging. However, post‐treatment, the absence of complications following surgery and radiotherapy sessions and the complete disappearance of disease symptoms have brought a sense of relief and optimism. The patient is now in the process of receiving prosthetic treatment, a significant step towards regaining normalcy and overall well‐being.

The management of synovial sarcoma involves a multidisciplinary approach due to its aggressive nature and potential for local recurrence and distant metastasis.^19^ Surgical excision remains the mainstay of treatment, aiming for complete resection with clear margins. However, the extent of surgery in the maxillofacial region may be limited by the proximity of vital structures. The rarity of synovial sarcoma in the maxillofacial region poses challenges in developing standardized treatment guidelines. The optimal therapeutic approach should be tailored to each individual case, considering factors such as tumor size, location, histological subtype, and the patient's overall health. Close collaboration between various specialties, including maxillofacial surgeons, pathologists, oncologists, and radiation therapists, is essential to provide comprehensive and personalized care to patients with synovial sarcoma in the maxillofacial region.

## 4 CONCLUSION

Synovial sarcoma in the maxillofacial region is an exceptionally rare entity presenting diagnostic and therapeutic challenges. Our case highlights the importance of considering synovial sarcoma as a differential diagnosis in patients presenting with maxillofacial tumors, even in young children. The accurate diagnosis and optimal management of synovial sarcoma rely on a multidisciplinary approach, incorporating clinical, radiographic, histopathological, and IHC evaluations. Further research and long‐term follow‐up are warranted to improve our understanding of this rare malignancy and refine treatment strategies.

## AUTHOR CONTRIBUTIONS

**Nasrollah Saghravanian:** Conceptualization; formal analysis; methodology; project administration; supervision. **Amin Rahpeyma:** Methodology; supervision; validation. **Mahsa Ghorbani:** Investigation; visualization; writing – original draft; writing – review and editing. **Pooya Saeedi:** Investigation; visualization; writing – original draft; writing – review and editing.

## FUNDING INFORMATION

This study was self‐funded and did not receive any financial assistance from any organizations.

## CONFLICT OF INTEREST STATEMENT

The authors declare no conflict of interest.

## CONSENT STATEMENT

Written informed consent was obtained from the patient's parents to publish this report in accordance with the journal's patient consent policy.

## Data Availability

The data that support the findings of this study are available on request from the corresponding author.
